# Affinity Labeling of Membrane Receptors Using Tissue-Penetrating Radiations

**DOI:** 10.1155/2013/503095

**Published:** 2013-06-27

**Authors:** Franklin C. Wong, John Boja, Beng Ho, Michael J. Kuhar, Dean F. Wong

**Affiliations:** ^1^Nuclear Medicine, University of Texas MD Anderson Cancer Center, Houston, TX 77030-4009, USA; ^2^Neuro-Oncology, University of Texas MD Anderson Cancer Center, Houston, TX 77030-4009, USA; ^3^Department of Radiology, Johns Hopkins Medical Institutions, Baltimore, MD 21205, USA; ^4^University of Texas MD Anderson Cancer Center, 1400 Pressler, Unit 1483, Houston, TX 77030-4009, USA; ^5^Addiction Research Center, National Institute of Drug Abuse, Baltimore, MD 21224, USA; ^6^U.S. Consumer Product Safety Commission, Bethesda, MD 20814, USA; ^7^Neuropharmacology, Yerkes National Primate Research Center of Emory University, Atlanta, GA 30322, USA; ^8^Radiology, Psychiatry, Neuroscience, Environmental Health Science, and Carey Business School, Johns Hopkins Medical Institutions, Baltimore, MD 21205, USA

## Abstract

Photoaffinity labeling, a useful *in vivo* biochemical tool, is limited when applied *in vivo* because of the poor tissue penetration by ultraviolet (UV) photons. This study investigates affinity labeling using tissue-penetrating radiation to overcome the tissue attenuation and irreversibly label membrane receptor proteins. Using X-ray (115 kVp) at low doses (<50 cGy or Rad), specific and irreversible binding was found on striatal dopamine transporters with 3 photoaffinity ligands for dopamine transporters, to different extents. Upon X-ray exposure (115 kVp), RTI-38 and RTI-78 ligands showed irreversible and specific binding to the dopamine transporter similar to those seen with UV exposure under other conditions. Similarly, gamma rays at higher energy (662 keV) also affect irreversible binding of photoreactive ligands to peripheral benzodiazepine receptors (by PK14105) and to the dopamine (D2) membrane receptors (by azidoclebopride), respectively. This study reports that X-ray and gamma rays induced affinity labeling of membrane receptors in a manner similar to UV with photoreactive ligands of the dopamine transporter, D2 dopamine receptor (D2R), and peripheral benzodiazepine receptor (PBDZR). It may provide specific noninvasive irreversible block or stimulation of a receptor using tissue-penetrating radiation targeting selected anatomic sites.

## 1. Introduction

Photoaffinity labeling utilizes UV photons to convert reversible binding between a photoreactive ligand and a receptor/protein into irreversible binding [1]. This biochemical technique has provided useful tools to characterize receptors and biologically important proteins, mostly *in vitro*, because the irreversible binding enhances the signal-to-noise ratio upon washout of the unbound ligand. The classic study of affinity labeling is the use of radiolabeled affinity ligand to bind membrane protein receptor followed by UV irradiation, washing, and radioactivity counts. The alternative is binding of nonradioactive photoaffinity ligand to membrane receptors, followed by UV irradiation, washing, and assay of residual available receptors. Free-radical formation is also the mechanism through which X-ray and gamma rays interact in biologic systems. In fact, interactions between low-dose of X-ray (20–100 cGy) and photoreactive substrates have been reported to result in >80% enzyme inhibition in a dose-related manner similar to the effects of UV [2].

Due to the poor tissue penetration of the UV photon, *in vivo* application of affinity labeling is limited. Although the exact mechanism of photoaffinity labeling is not clear, free-radical formation is inevitably involved. Therefore, if the photoreactive ligand can be activated and converted into an irreversible bound label by tissue-penetrating radiation, it may prove useful in *in vivo* studies. The translation from *in vitro* to *in vivo* studies could be started with established membrane neuroreceptor systems. When established, neuroreceptor and other affinity labeling systems can be measured *in vivo* by advanced tomography including PET and SPECT.

Striatal dopamine transporter (SDT), which mediates the actions of many drugs, has been investigated using PET for diseases including drug abuse. These studies have utilized high-affinity reversible ligands. Several photoreactive ligands (including the three studied in this project) have been developed as well [3].

In baboon and human brains, striatal dopamine-2 receptors (D2R) can be imaged and quantified by PET using ^11^C-N-methylspiperone, NMSP [4]. The density of D2R is found to be elevated in the prolactin secreting human pituitary adenomas [5] and nonsecreting macroadenoma [6]. The D2R photoaffinity labeling agent, azidoclebopride, is commercially available and has been used to irreversibly label the D2R [7, 8]. Since PET studies of human D2R are well-established, it may be interesting to study these compounds before and after irradiation.

Most abundantly found in the kidneys, peripheral benzodiazepine receptors (PBDZR) localize in the mitochondrial membranes with porphyrins as the endogenous ligand. It is involved in the depression of the respiration ratio and oxidative phosphorylation; *in vitro* studies show that PBDZR ligands stimulate proliferation of the neoplastic cells and inhibit reproduction of the normal immune cell [9]. The levels of PBDZR are shown to be markedly elevated (3–20-folds) in animal gliomas, human gliomas, and colonic and ovarian carcinomas. Positron emission tomography using ^11^C-PK11195, a PBDZR ligand, was able to distinguish gliomas from normal brain tissue in *in vivo* human studies [10, 11]. The potential of PBDZR photoaffinity labeling is best illustrated with PK14105 which if unbound has a favorable rapid dissociation feature [12]. This feature is desirable because the difference between the irreversible ligand bond and reversibly bound residual ligand may be maximized by subsequent washout of the free ligand over time. PK14105 has also been found to serve a good potential tracer to evaluate brain lesions [11] as well as immune tissues in rats [13].

X-ray and gamma rays are identical forms of higher energy photon radiations differing only by their origins. X-ray is produced by machine while gamma rays are produced by radionuclides. Direct inactivation of enzymes by ionizing radiations such as target-site analysis occurs at higher doses (>1000 Gy). On the other hand, radiochemical reactions in biologic systems are predominantly mediated by free-radicals formation at lower radiation doses (e.g., <10 Gy). Therefore, the plausibility of specific and irreversible affinity labeling is studied using these tissue-penetrating radiations, X-ray, and gamma rays which are ionizing radiation. Besides conventional ionizing radiations, higher doses of ultrasounds at therapeutic range have been observed to alter *in vitro* enzyme kinetics [14] and affect cell damages [15]. Synergistic damaging effects have been reported with high-dose ultrasounds when hematoporphyrins were used to treat sarcomas [16]. Plausible mechanisms involve free-radical formation [17] and/or singlet oxygen formation [18]. The potential common mechanism of free-radical formation in photoaffinity labeling, X-ray/gamma ray, and ultrasounds should be further investigated.

The present *in vitro* study investigates the effects of X-ray on the irreversible binding to the striatal dopamine transporter (SDT) using three different photoreactive affinity ligands, RTI-38, RTI-63, and RTI-78. In a parallel set of *in vitro* experiments, we study the irreversible binding to the peripheral benzodiazepine receptors (PBDZR) and dopamine-2 receptors (D2R) with two photoreactive affinity ligands, respectively. The characterization of affinity labeling of receptors using tissue-penetrating radiation will provide a foundation for the development of receptor-specific or tissue-specific delivery of extrinsic diagnostic or therapeutic agents.

## 2. Materials

The three RTI compounds, p-azidobenzoylecgonine methyl ester tartrate (RTI-38), 3-*β*-benzoyloxy-8-methyl-8-azabicyclo [3.2.1] octan2-carboxylic acid p-azidophenyl ester hydrochloride (RTI-63), and 3-*β*-(p-chlorophenyl) tropan-2-*β*-carboxylic acid p-azidophenylethyl ester hydrochloride (RTI-78), were supplied by Carroll [3, 19]. [^3^H]-CFT, [^3^H]-NMSP, [^3^H]-N-methyl-PK 11195 (NET885), and nonlabeled PK11195 were supplied by New England Nuclear (Boston, MA). The nonradioactive PK14105 was a gift from Rhone-Poulenc Rorer (France).

## 3. Methods

### 3.1. Membrane Receptor Preparation for D2R and PBDZR Binding

Male rats weighing from 200 to 250 g were used for D2R experiments. The animals were decapitated, the brains were immediately removed, and the striatum was excised and stored at −70°C until used. Dog kidney membranes were similarly prepared for PBDZR experiments and stored. Tissue preparation was carried out as described by Kleven et al. [20]. The tissue was homogenized briefly in 20 volumes (w/v) of 50 mM Tris-HCl, pH 7.4, with 5 mM Na-EDTA and 50 mM NaCl at 4°C. The homogenate was centrifuged at 48,000 ×g for 15 min at 4°C. The resultant pellet was rehomogenized in Tris-HCl buffer (with 50 mM NaCl) and centrifuged again. The final pellet was resuspended in 50 mM Tris-HCl buffer with 100 mM NaCl, pH 7.4, to a final concentration of 5–10 mg tissue/mL or 2.5 to 3.0 mg protein/mL.

### 3.2. Irradiation

Using UV irradiation, aliquots of tissue suspension were incubated in the dark with the photoaffinity ligand azidoclebopride (1 mM final concentration) for D2R and PK14105 for PBDZR for 90 minutes at room temperature. A 15-mL tissue suspension in an uncovered plastic petri dish (100 × 13 mM) (fluid depth, 3-4 mM) was irradiated 11 cm away from a light source (ultraviolet lamp) for 30 seconds. 

For X-ray irradiation, a Dynamax 42–40 rotating-anode X-ray tube unit (Machlett Laboratories, Inc., Stamford, CT, USA) set at 115 kVp and 180 mAs was used to treat receptor preparations in test tubes with repeated exposures; the radiation dose was calibrated by thermoluminescent dosimeters.

For gamma ray irradiation, a NASATRON (F0103) irradiator (US Nuclear Co.) (662 keV) from Cesium 137 source was applied to receptor preparations in test tubes covered with aluminum foil. The calibrated dose rate range was from 1 to 10 Gy/minute. The total radiation dose was determined from the dose rate and the exposure time.

Ultrasound irradiation experiments were briefly conducted and involved the use of a calibrated sonicator (Sonicator II, Model ME702, 10 cm crystal, 1 MHz, Mettler Electronics, Anaheim, CA, USA) with the applicator immersed in ice-cold water irradiating the test tube (1 cm in diameter) containing PK14105 and dog kidney membrane preparation (prepared and stored in similar manners as with the rats membranes). It was turned on at a single intensity setting of 1 MHz at 2 Watt per sq. cm for 2 and 10 minutes, respectively.

### 3.3. Binding Assays and Data Analyses

For SDT binding, striatal membranes were first incubated with (50–100 uM) of photoreactive ligands (RTI-38 and RTI-63) in the presence or absence of 25 uM cocaine. Higher dose of cocaine (50 uM) was used with the higher affinity agent RTI-78. The IC_50_ for RTI-38, RTI-63, RTI-78, and cocaine has been determined to be 475, 227, 6, and 102 nM, respectively. They were then irradiated with UV or X-ray. After 5 cycles of wash with buffer and centrifugation, the SDT was assayed as described above. The concentration of the radioactive ligand ^3^H-CFT was 50 pM.

For dopamine D2R binding assays, samples (1.0 mL) containing striatal membrane suspension (0.05–0.12 mg protein/mL) in 50 mM Tris-HCl buffer were incubated for 45 minutes (37°C) with [^3^H]spiperone (from 0.08 to 0.1 nM) in the presence or absence of (+)butaclamol (10 mM) for nonspecific binding [21]. For peripheral PBDZR binding assays, kidney homogenate (0.05–0.2 mg protein in 0.5 mL) was incubated with [^3^H]-PK11195 (1 nM). Nonspecific binding was determined by the addition of PK11195 (1000 nM) [22]. Membrane-bound striatal ligands were separated by filtration through Whatman GF/B filters followed by two washes with 5 mL of cold incubation buffer. The radioactivity on the filters was assayed by liquid scintillation spectrometry.

**Figure 1:**
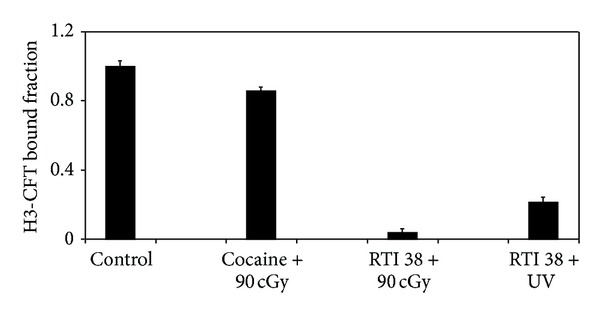
Affinity labeling of SDT with 50 uM of RTI-38 using UV or X-ray. Irreversible and specific binding of RTI-38 to SDT is noted with 90 cGy of X-ray or with UV (*F* = 840, *P* < 0.001). *t*-tests and one-way ANOVA indicate statistically significant difference from the control group (*t* = 39.5, *P* = 0.010) or the cocaine group treated with X-ray (*t* = 45.7, *P* = 0.009).

**Figure 2:**
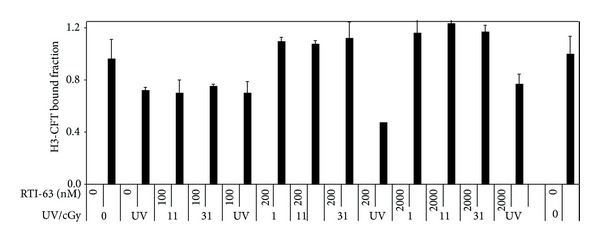
Affinity labeling of SDT with RTI-63 using UV or X-rays. While UV affects affinity labeling of SDT (*F* = 10.4, *P* = 0.009) in a manner related to RTI-63 concentration (*F* = 6.7, *P* = 0.009), no significant dose-related binding is identified with respect to X-ray dose (11–31 cGy) or to RTI-63 concentrations (100–2000 nM). The largest magnitude of gamma-rays-induced binding occurs with RTI-63 at 100 nM (*t* = 7.0, *P* < 0.001).

**Figure 3:**
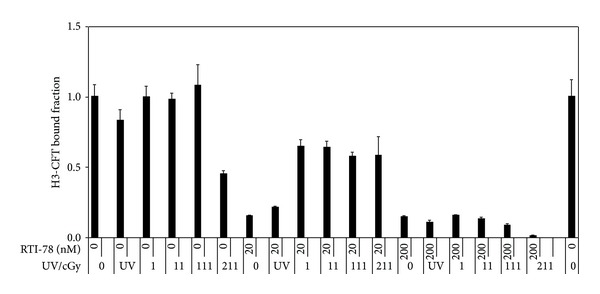
Affinity labeling of SDT with RTI-78 using UV or X-ray. Binding data suggest possible dose-related binding with respect to radiation doses as well as RTI-78 concentration. While one-way ANOVA indicates significance (*F* = 208.6 and *P* < 0.001) for UV for both 20 nM and 200 nM RTI-78, two-way ANOVA indicates significance for both doses of X-ray (*F* = 32.8 and *P* < 0.001) and concentration of RTI-78 (*F* = 535.7 and *P* < 0.001) with interaction (*F* = 28.0 and *P* < 0.001).

**Figure 4:**
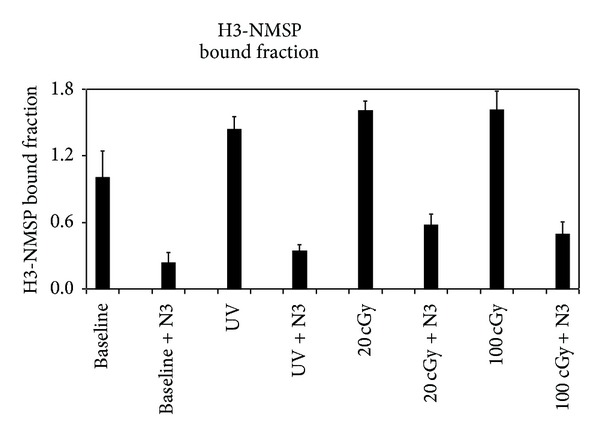
Affinity labeling of D2R with azidocleopride (N3) using UV or gamma rays. Irreversible and specific binding to peripheral benzodiazepine receptor, D2R, is noted after exposure of the mixture of D2R and 1 uM N3 under UV or gamma rays. Statistically significant correlations of binding to N3 (*F* = 27.3, *P* < 0.001) and gamma rays doses (*F* = 4.3, *P* = 0.04) are revealed by two-way ANOVA.

**Figure 5:**
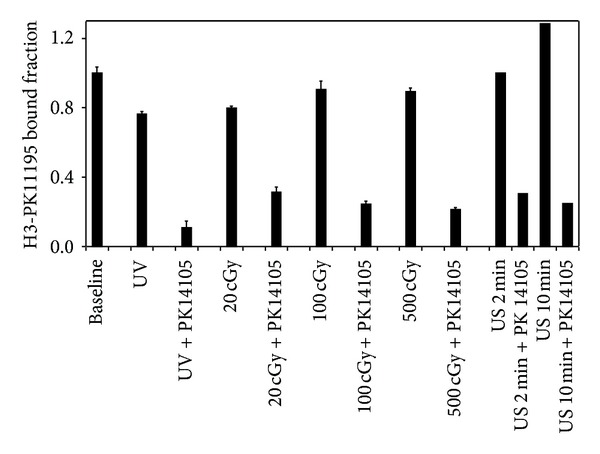
Affinity labeling to PBDZR with PK14105 using UV, gamma rays, or ultrasounds. Affinity binding of PK14105 to PBDZR under UV, gamma rays, and ultrasounds is confirmed. Two-way ANOVA was used to analyze all data found significant for gamma rays doses (*F* = 4.1 and *P* = 0.011) and PK14105 (with *F* = 482.0 and *P* < 0.001). Ultrasonic exposure (2 Watts/sq. cm at 1 MHz) of the reactants on ice in the dark for 2 and 10 minutes affects binding of 69% and 80%, respectively, in single-point experiments.

The extent of irreversible binding of the nonradioactive affinity labels to the receptors was calculated as the decrease in the subsequent binding with [^3^H]-labeled ligand. Fractions of bound radioactive ligand were tabulated and analyzed using one-way ANOVA and two-way ANOVA from SigmaStat (version 3.5, Systat software, Point Richmond, CA, USA). Data and standard errors are plotted with Microsoft Excel 2003 graphics options (Microsoft Inc., Redmond, WA, USA) in Figures 1–5. The Holm-Sidak pairwise-comparison option was adopted to obtain *t*-test scores.

## 4. Results

### 4.1. X-Ray Affinity Binding of RTI-38, RTI-78, or RTI-63 to SDT (Figures 1, 2, and 3)

Striatal membranes prepared as described in Section 3 were incubated with 0.05 mM of RTI-38, 20–200 nM of RTI-78, or from 100 nM to 2000 nM of RTI-63 for 30 minutes on ice. The mixture was irradiated with total doses from 1 to 500 cGy X-ray. Binding experiments using 90 cGy were able to achieve 96% binding with RTI-38 while a concurrent UV experiment achieved 79% inhibition of binding, both of which are different from the control group or cocaine under X-ray without RTI-38 (Figure 1). One-way ANOVA confirmed statistical significance with *F* = 840.3 and *P* < 0.001 and all treatment groups. Although the small but significant control group of cocaine with 90 cGy X-ray might need to be further explored, these RTI-38 experiments at high reactant concentration (>1000 nM) were not further pursued because the limited supply was exhausted. 

Although RTI-63 in combination with UV significantly decreased subsequent CFT binding (two-way ANOVA with *F* = 10.4 and *P* = 0.009 for UV and *F* = 6.7 and *P* = 0.009 for RTI-63), no dose-related response is noted (Figure 2). Large variations are noted in the baseline binding under X-ray without RTI-63 and may be related to technical variance, in light of subsequent similar baseline binding of CFT close to unity with X-ray up to 111 cGy in Figure 3. Although significant binding is noted with 100 nM of RTI-63 (*T* = 7.0, *P* = 0.009), the lack of dose-related responses to RTI-63 levels or to X-ray doses indeed brings into doubt the utility and efficiency of RTI-63 as X-ray affinity labels.

Initial experiments indicated that reversible inhibition of SDT occurs with RTI compounds at nM range and the initial experiment with RTI-38 used concentrations that were supersaturating to the SDT. Subsequent experiments were carried out at lower concentrations. RTI-78 at 20 nM inhibited specific binding (36%–43%) with X-ray, but no dose-response relationship was identified. With the higher dose of RTI-78 at 200 nM, dose-related specific irreversible binding (85%–99%) is demonstrated with increasing radiation dose from 1 cGy to 211 cGy (Figure 3). While one-way ANOVA indicates significance (*F* = 208.6 and *P* < 0.001) for UV for both 20 nM and 200 nM RTI-78, two-way ANOVA indicates significance for both doses of X-ray (*F* = 32.8 and *P* < 0.001) and concentration of RTI-78 (*F* = 535.7 and *P* < 0.001). Interaction between X-ray and RTI-78 was also noted preventing identification of the main effect (*F* = 28.0 and *P* < 0.001).

### 4.2. Gamma Ray Affinity Labeling of D2 Receptor (Figure 4)

When using 1000 nM of azidoclebopride (N3), specific and irreversible D2R binding was induced by gamma rays in a dose-related manner. In spite of moderate variations in the bound fractions of baseline groups and the baseline group with N3, two-way ANOVA found significant correlation of specific binding with gamma ray doses (*F* = 4.3 and *P* = 0.042) and with N3 (*F* = 27.3 and *P* < 0.001). Gamma rays of 20 cGy were able to affect significant binding above baseline (*T* = 2.9 and *P* = 0.019). UV irradiation induced 34% specific binding (one-way ANOVA, *F* = 11.6 and *P* = 0.009). This latter figure is similar to previously published results [7, 8]. 

### 4.3. Affinity Labeling of PBDZR Using Gamma Rays (Figure 5)

While the effects of UV on binding are not different than those of control, UV in combination with PK14105 is significantly different from UV alone or control (one-way ANOVA findings of *F* = 566.7 and *P* < 0.001; *T* = 29.3 and 29.0, resp.). Irreversible and specific binding is noted at 60.8%, 72.3%, and 76.2% using 20 cGy, 100 cGy, and 500 cGy of gamma rays, respectively. Two-way ANOVA found significant data for gamma rays doses (*F* = 4.1, *P* = 0.011) and PK14105 (*F* = 482.0 and *P* < 0.001), respectively. A weak dose-related response was also noted with PK14105 between 50 and 500 cGy (*T* = 3.9 and 3.6, resp., for 50 cGy versus 100 cGy and 500 cGy, resp.).

## 5. Discussion

We have demonstrated that photoaffinity labeling of photoreactive ligands using UV may be selectively extended to using tissue-penetrating radiations including X-ray, gamma ray, and possibly ultrasound. However, even with the same receptor system (e.g., SDT), this extension is not observed to the same degree with all photoaffinity labels (e.g., not with RTI-63) and needs to be confirmed for each compound individually. Although no direct mechanism is proposed to explain radiation-affinity labeling, the most likely common underlying process would be free-radical formation to affect covalent binding of photoreactive moieties in the affinity label to the neighboring receptor/protein. Therefore, in the selection of compounds for radiation-affinity labeling experiments, photoaffinity labels will serve as good starting points. 

Low-dose (<100 cGy) X-ray (250 kVp) irradiation of photoreactive substrate to cause enzyme inhibition has been reported by a single study [2]. The end point of this latter study is inhibition of enzyme activity, which is a functional measure and subjected to other regulatory factors (cofactors or other substrates) that may also be altered by X-ray. In this affinity binding study, radiation of higher energy (e.g., X-ray and gamma ray) as well as lower energy radiation (e.g., ultrasound) achieves immediate and direct labeling. Therefore, arguably, affinity labeling is a more direct and more efficient method to evaluate the effects of the radiation. Furthermore, this affinity labeling mechanism may allow selective and locoregional delivery of photoreactive drugs to stimulate or block receptors.

Affinity labeling using UV has been used to study “*in vitro*” chemistry. Affinity labeling using X-ray, gamma ray, or ultrasounds may allow study of “*in vivo*” chemistry. Further investigations of radiation-affinity labeling may provide new ways to produce better molecular imaging or drug delivery. For instance, if nonradioactive PK14105 is injected during brain radiotherapy, the concurrent binding of this drug to the brain cortices may be visualized by subsequent PET imaging using ^11^C-PK11195 according to established procedures in humans as by Junck et al. [10]. Alternatively, ^18^F labeled-PK14105 may be synthesized as described [21] and directly injected before or during scheduled radiotherapy of organs rich in PBDZR (such as brain lesions or kidney); the paths and amounts of radiation absorbed may be visualized. The PBDZR has been identified to a translocation protein (TSPO) which further pinpoints functional roles of PBDZR pathways and have gained increasing interest in molecular biology [23, 24].

^11^C-NMSP may be similarly used to study binding of photoreactive D2R drugs such as azidoclebopride during or before radiotherapy to evaluate radiation effects. The baseline variation with azidoclebopride alone (Figure 4) in the dark showed irreversible binding that is different from those in the literature [7, 8] and needs to be further studied. The rising binding with UV and increasing gamma ray doses above baseline fraction of 1.0 also needs further investigation. Plausible mechanisms include destruction of internal bound ligands releasing or unfolding receptor to be bound by subsequent ligands. In spite of these variations, there is statistical significance of UV and gamma ray to affect irreversible-specific receptor binding between azidoclebopride and D2R.

Ultrasounds at higher intensity (0.5 W for 10 minutes) have been found to produce free-radicals and affect radical-related therapeutic effects [15, 16]. To test the ability of ultrasound to affect affinity labeling with PBDZR, ultrasound exposure (2 Watts/sq. cm at 1 MHz) of reactants on ice in the dark for 2 and 10 minutes affects irreversible binding of 69% and 80% in two experiments of single data point (Figure 5). The radionuclide supply and membrane preparations were exhausted during these experiments, and preliminary findings with ultrasounds need to be verified. If confirmed, may be found wider *in vivo* applications because of the wide availability of ultrasound instruments and no ionizing radiation is involved. 

This *in vitro* qualitative study is a survey of performing irreversible affinity labeling of readily available receptor systems (rat SDT, rat D2R, and dog kidney PBDZR) using available irradiation sources (X-ray, gamma, and ultrasound) for proof of concepts. It has illustrated the possibilities of radiation-affinity labeling. The concentration of study compounds is in the nano- to micromolar range and is within pharmacologic range following systemic administration. The current study examines the interaction of photoaffinity labels with radiation at dose ranges feasible in the clinical setting. The gamma ray radiation doses (1–200 cGy) are within ranges of external beam radiotherapy for cancers (total 2000–6000 cGy or fractionated doses of 100–300 cGy daily). Ultrasound (2 W/min for 2 to 10 minutes) strength is within range of routine therapeutic use in rehabilitation medicine. Therefore, large fractions/quantities of photoreactive chemicals may be delivered to target tissues at selected time using switchable external irradiation commonly used in humans with cancer or ultrasounds. When further *in vitro* and animal experiments verify this concept, tissue-penetrating radiation-affinity labeling may be translated to the clinics.
